# Factors associated with nursing students’ medication competence at the beginning and end of their education

**DOI:** 10.1186/s12909-015-0513-0

**Published:** 2015-12-18

**Authors:** Virpi Sulosaari, Risto Huupponen, Maija Hupli, Pauli Puukka, Kirsti Torniainen, Helena Leino-Kilpi

**Affiliations:** Department of Nursing Science, University of Turku, FI-20014 Turku, Finland; Clinical Pharmacology Unit, Department of Pharmacology, Drug Development and Therapeutics, University of Turku, FI-20014 Turku, Finland; National Institute for Health and Welfare, PL 57, FI-20521 Turku, Finland; Hospital Pharmacy Hospital Pharmacy, Turku University Hospital, PO Box 52, FI-20521 Turku, Finland

**Keywords:** Nursing student, Medication competence, Competence, Medication calculation skills, Pharmacological skills, Nursing education, Learning style

## Abstract

**Background:**

In previous studies, deficiencies in nursing students’ medication competence have been highlighted. However, the focus of research has been limited especially to medication calculation competence and factors associated with it. In order to develop undergraduate nursing education and research, an understanding of the individual and learning environmental factors associated with medication competence from a broader approach is warranted. Our aim was therefore to evaluate the theoretical, practical and decision-making competence of nursing students and to identify factors associated with their medication competence at the beginning and end of their education.

**Methods:**

We used a descriptive, correlational study design with a structured instrument including a set of potential associated factors, knowledge test, medication calculation test and patient vignettes. The participants were nursing students at the beginning (*n* = 328) and at the end of their education (*n* = 338). Data were analyzed statistically.

**Results:**

In the evaluation of theoretical medication competence, the students’ mean score over the semesters was 72 % correct answers in a knowledge test. In the evaluation of practical medication competence, the mean score was 74 % correct answers in a medication calculation test. In the evaluation of decision-making competence, the mean score was 57 % correct answers on deciding the best action in the situation given in patient vignettes. At the end of their education, students were able to solve patient vignettes significantly better. Individual factors were most evidently associated with medication competence. At the beginning of their education, students’ previous academic success had a stronger association with medication competence. However, at the end of the education students’ abilities in self-regulated learning and study motivation were more significant factors.

**Conclusion:**

The core elements of medication competence are significantly interrelated, highlighting the need to provide integrated and comprehensive medication education throughout the undergraduate education. Students’ learning style is associated with medication competence. There is a need for methods to identify and support students having difficulties to self-regulate their learning. To increase the safety of medication care of patients, research focusing on the development of effective teaching methods is needed. This study produced information for future nursing education research in this field, especially for interventional studies.

**Electronic supplementary material:**

The online version of this article (doi:10.1186/s12909-015-0513-0) contains supplementary material, which is available to authorized users.

## Background

Medication management is a complex and high-risk activity forming a major part of registered nurses’ responsibilities in their everyday practice [1, 2]. It involves professional tasks starting from identifying the need for medication use, ordering, storage, safe handling and preparation of medication for and administration to patients, monitoring and evaluating the effectiveness of treatment, as well as documentation and patient education [38]. For this professional activity nurses need to have adequate medication competence. Therefore, the goal of undergraduate education is to provide opportunities for students to become competent nurses [3, 4].

In this study, the concept of medication competence is based on an integrative literature review by Sulosaari et al. [38]. Medication competence requires a solid knowledge base in pharmacology, pharmacy and medication management (theoretical medication competence). In addition, the student must be able to apply that knowledge in real-life situations (practical medication competence). Decision-making competence combines theoretical and practical competence and refers to students’ competence in making decisions on patients’ medicine regimens.

In undergraduate nursing education, attention needs to be focused on nurses’ educational preparation since similar deficiencies in nursing students’ medication competence have been highlighted [5, 6–11]. However, most previous studies have been targeted at students’ medication calculation and numeracy skills, although calculation errors are only one factor contributing to medication errors [12]. Only few studies have evaluated medication competence from a broader perspective [10, 11, 21].

### Nursing students’ medication competence

Both students’ numeracy and medication calculation skills have been found to be insufficient, giving rise to major concern over the issue [7, 9–11, 13–20]. In some studies, mathematical and medication calculations have been mixed in the test, making interpretation of the results difficult. In earlier studies, the average share of correct answers on calculation tests seems to be around 60 % correct answers, ranging from 35 % in the McMullan et al. [9] study to 71 % in the Simonsen et al. [11] study. A minority of students achieve a perfect result with 100 % correct answers. Previous research indicates deficiencies in the students’ pharmacological knowledge base, with the average score ranging from 55 % [10] to 71 % [6, 11] correct answers on a knowledge test. Recently, Simonsen et al. [11] found deficiencies in nursing students’ knowledge of drug management, with an average score of 43 % correct answers.

### Factors associated with medication competence of nursing students

Individual and learning environment factors related to the clinical practice and educational institutions are important determinants of medication competence [21]. In the previous studies, the individual factors associated with nursing students’ medication calculation competence have been students’ age [7, 9, 15], previous academic success [7, 13, 15, 16, 18, 20, 22], self-confidence or self-efficacy in mathematics [4, 23–25], the phase of nursing education [7] and being an international student [4]. Certain approaches to learning have been associated with better academic performance [26]. Nursing students have also perceived their learning style to be important in learning medication care [27].

Learning environments within health care education have evolved significantly over the years. Nursing curricula are often over-loaded with content, and e-learning has become increasingly accessible, posing a challenge for the students to cope with. The time spent on self-directed learning, referring to students’ ability to manage and control their own learning [3, 27, 29, 30] is increasing at the expense of traditional classroom education. To regulate their learning activities, students may let themselves be directed by external sources, or they may direct themselves to be able to acquire new knowledge and solve new problems independently [35].

Of the environmental factors contributing to learning, clinical practice within health care institutions has been highlighted by students as important for the development of medication competence [27]. The clinical practice environment allows them to understand the effects of medical treatment in real life [31] and to practice decision-making in medication care [32]. However, according to students’ experiences, there are too few learning opportunities available in clinical practice [27, 32]. An environmental factor under scrutiny among nurse educators is the use of calculators in medication calculations [8]. The use of a calculator does not improve results in numeracy or medication calculation tests if the student’s problems are conceptual. However, the use of a calculator prevents arithmetic errors [19].

Since research on students’ theoretical competence in applying their knowledge into practice and making decisions to solve problems with patients’ medicine regimens is largely lacking we decided to approach nursing students’ medication competence from these perspectives. We therefore evaluated students’ theoretical and practical medication competence and decision-making at the beginning and at the end of their studies. In this study, the evaluation of practical medication competence is, however, limited to students’ medication calculation competence.

### Aim

The aim of this study was to evaluate the theoretical, practical and decision-making medication competence of nursing students at the beginning and at the end of their nursing education and to identify the factors associated with it. The results can be used to develop undergraduate medication education and research. More precisely, we a) evaluated the theoretical, practical medication and decision-making competences of the students, b) evaluated the overall medication competence of the students, and c) identified factors associated with medication competence at the beginning and at the end of their education.

## Methods

### Participants

This descriptive, correlational study examined students’ medication competence and associated factors with two samples of nursing students at the beginning (2nd semester, *n* = 328) and end (7th semester, *n* = 338) of their education in 12 out of 23 polytechnic schools in Finland, representing different geographic areas and different school sizes. Collecting the samples at two different stages of the curriculum enabled us to identify the changes in medication competence during education and to explore potential differences between the factors associated with it in the two groups. The 2nd semester students had participated in basic education in pharmacology and medication management but had limited experience from clinical practice.

The participants were bachelor-level students with an undergraduate program of 3.5 years, corresponding to 210 ECTS credits in the European Credit Transfer and Accumulation System [ECTS]. The program is a full-time course with seven semesters, one ECTS credit corresponding to 27 working hours on the part of the student [European commission http://ec.europa.eu/education/ects/ects_en.htm]. As guided by European Regulations, approximately half of the education is in clinical practice. The length and number of clinical practice periods varies between the schools. However, the average is 7 to 8 clinical practice periods during undergraduate education. In clinical practice students are gradually exposed to increasingly more complex scenarios in the clinical setting, such as patients with multiple medications and co-morbidities. Students use a learning workbook entitled “Medication Passport” during their clinical practices. The Medication Passport includes learning tasks on medication management. A more detailed description of medication education in Finland has been published in another article [5].

### Data collection

Data were collected over an 18-month time period in 2011–2012, with either an electronic or a paper version of the questionnaire. Students received information about the study either by e-mail or from a teacher in the classroom (*N* = 1314). Permission to use a calculator for medication calculations was given to some of the students for the purpose of evaluating the association between the use of a calculator and the results on the medication calculation test [9]. We aimed at achieving a minimum of 100 students using calculators (10 % of the sample). In the final sample, 19 % (*n* = 126) of the students had a calculator in use. The students had up to 90 min for answering.

The total response rate was 51 % (*n* = 666). At the time when the study took place, there were nearly 2,000 nursing students in the 2nd and 7th semester. Sample size calculations with significance level 0.05 and power 0.80 were based on the data in the study of Grandell-Niemi [33] and our pilot study (*n* = 69), giving the minimum number of 300 students in both groups.

### Questionnaire design

The selection and development of the questionnaires used in this study was conducted on the basis of two literature reviews [21, 38] and a multidisciplinary expert panel (n = 10). The data were collected with the Medication Competence and Associated Factors [MCAF] instrument developed for the study. Some items from other instruments [33, 34] were integrated into MCAF. The evaluation of theoretical medication competence consisted of four subcategories: (a) legislation and guidelines, (b) medication package information and common abbreviations used, (c) pharmacology, and (d) handling and preparation of medications ready for use and medication administration. The practical medication competence was evaluated by ten medication calculations. The evaluation of decision-making competence consisted of ten patient vignettes. Examples of the instrument section on evaluating students’ medication competence are available in Additional file 1.

For exploring students’ learning styles, part of the Learning Style Inventory [ILS] was used [34]. The part we used had been previously validated in Finland with nursing and medical students [36, 37]. The ILS instrument consisted of three sum scores: Self-Regulation, External Regulation, and Lack of Regulation in learning.

**Fig. 1:**
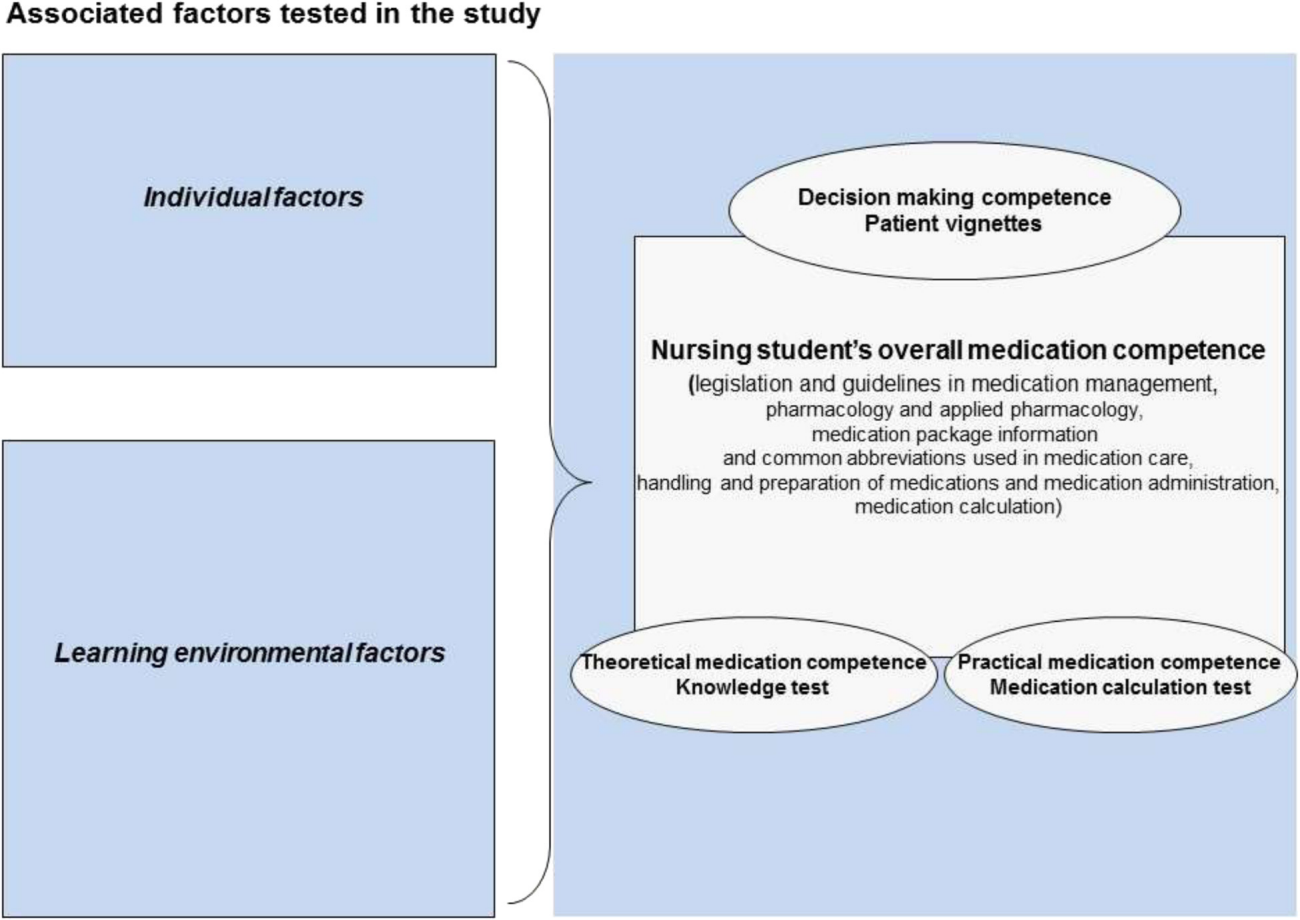
Framework of the study

A set of potentially associated factors was tested in relation to medication competence (Fig. 1). Individual factors were socio-demographic factors, educational background and academic success, active participation in medication education, motivation for studying medication education, self-confidence in medication administration, satisfaction with medication education, work experience and learning style. The learning environmental factors were the number of clinical practice placements, semester, the polytechnic school, the use of the Medication Passport and the use of a calculator.

### Data analysis

The data were analyzed using Statistical Package for Social Sciences (version 20) and SAS for Windows (version 9.1). Descriptive statistics was used to summarize the information gathered in the survey. Missing values were excluded from the analysis. On the potential associated factors, sum scores were formed for Self-confidence in Medication Administration (6 items), Active Participation in Studying Topics of Medication Care (4 items), Self-regulation (ILS, 5 items), External Regulation (ILS, 5 items), and Lack of Regulation in Learning (ILS, 4 items). On the evaluation of medication competence, sum score for the analysis consisted of Medication Competence (50 items), and sub-sum scores of Theoretical Medication Competence (30 items), Practical Medication Competence (10 items) and Decision-making Competence (10 items). The response "I don’t know" was coded as a wrong answer. The students received one point for each correct answer. In six of the patient vignettes, there was only one correct answer, and in four cases there was one best choice for action (coded as two points), while the second best choice was coded as one point. After coding incorrect answers and 'I don’t know' answers as 0, the proportion of correct answers was calculated for all sum scores on medication competence.

Depending on data distribution, comparisons between groups were analyzed with Chi-square or Fishers exact test; *t*-test or Mann–Whitney *U*-test; ANOVA or Kruskal-Wallis. To explore the interrelationships between interval variables, Pearson and Spearman correlation analysis was used. Two-tailed significance tests were used, and *p*-value <0.05 was considered statistically significant. The normality of distributions was tested by Shapiro-Wilk test. All numerical variables used in regression analysis were close enough normally distributed for this parametric method. Finally, standard multiple regression was applied to determine the presence of any statistically significant explanatory variables.

### Reliability and validity

The MCAF instrument was developed for the study. In addition, we used items from previously validated instruments [33, 34] as part of the MCAF. The ILS instrument has been validated previously [35–37]. The content-related validity of the instrument was good, as assessed on the basis of the systematic process of instrument development, including two systematic reviews [20, 37], an expert panel (*n* = 10) and the pilot study (*n* = 69).

The internal consistency of the instruments showed adequate reliability in all dimensions of the MCAF and ILS instrument (Cronbach’s α 0.70 – 0.80), with the exception of the External Regulation sum score in the ILS instrument (Cronbach’s α 0.52). The External Regulation in Learning score showed lower reliability than in previous studies [36, 37] and needs further validation.

### Ethical aspects

Ethical approval was given by the Ethical Board of the University of Turku, and the permission for the study was given by all of the polytechnic schools. Participation was voluntary and anonymous. Confidentiality of the data was assured. All participants were given information about the study and its purpose. Answering the questionnaire was seen as consent to participate in the study. Permission to use previously developed instruments was given before the data collection.

## Results

Of the 2nd semester students, 88 % were female and 12 % male. Of the 7th semester students 90 % were female and 10 % male. With the 2nd semester students ages varied between 19 and 55 years and with the 7th semester students between 21 to 51 years. Mean age was 25 years in the two groups. Most of the students had completed upper secondary school (65 %) and a short syllabus in mathematics (41 %). 22 % of the students had a prior degree in nursing. Most of the students (64 %) had failed the medication calculation test at least once during their studies, and 20 % had participated in supportive education in medication calculation. Supportive medication calculation education most often refers to a teacher-led workshop with additional practice in medication calculation. The 2nd and 7th semester students had similar educational backgrounds (Additional file 2).

On average, the 2nd semester students had only one clinical practice period while the 7th semester students had seven. There were some differences between the two groups in their perceptions of learning medication care, self-confidence and learning style (Table 1). The 7th semester students were less satisfied with the amount of medication education and perceived themselves to be more active and more confident than the 2nd semester students. The 2nd semester students had a more externally regulated learning style in their studies and lack of regulation in learning. In general, the students demonstrated a low level of abilities in Self-Regulation in Learning (ILS).

**Table 1:** Students’ perceptions on learning medication care, self-confidence and learning style

	2nd semester (*n* = 328) Mean (SD)	7th semester (*n* = 338) Mean (SD)	Difference between the groups (*p*-value)
A) Learning medication care (Scale 1–5, strongly disagree–strongly agree)			
Perceives mathematics easy	3.33 (1.15)	3.33(1.13)	ns
Perceives medication calculations easy	3.49 (1.03)	3.55 (0.97)	ns
Perceives pharmacology easy	2.76 (0.85)	2.69 (0.83)	ns
Satisfied with current amount of medication education	2.88 (1.03)	2.44 (1.07)	<0.001
Good motivation for studying medication care	4.06 (0.76)	3.98 (0.80)	ns
B) Active participation in studying topics of medication care (Sum-score on a scale of 1–5, strongly disagree–strongly agree)	3.71 (0.61)	3.81 (0.68)	0.017
C) Self-confidence in medication management (Sum-score with scale 1–5 strongly disagree-strongly agree)	3.43 (0.55)	3.66 (0.60)	<0.001
D) Learning style (ILS) (Sum-scores on a scale of 1–5: Never–always)			
Self-regulation ^1)^	2.37 (0.78)	2.28 (0.81)	ns
External regulation ^2)^	3.41 (0.59)	3.18 (0.61)	<0.001
Lack of regulation ^3)^	2.09 (0.66)	2.02 (0.77)	0.03

*ns* no statistical difference, *SD* Standard deviation, Statistical test: Mann–Whitney *U*-test,^1)^ The higher the score, the better the students’ ability in self-regulating learning, ^2)^ the higher the score, the more external regulation in learning, ^3)^ the lower the score, the less the student has lack of regulation in learning

### Medication competence of the nursing students

#### Theoretical medication competence

None of the students achieved 100 % correct answers in the knowledge test (Additional file 3); the mean percentage of correct results over the semesters was 72 %. The difference between the two groups was statistically significant only in medication package information and common abbreviations used in medication care (*p* < 0.001).

### Practical medication competence

All ten medication calculation tasks were calculated correctly by 17 % of the students; the mean result over the semesters was 74 % correct (Additional file 4) with no statistically significant differences between them. Some items were managed better by students at the end of their education than by their younger colleagues; this was true for dilution calculations, converting units of infusion liquids from 5 % strength to mg/ml. On average, 85 % correct results (mean 8.66/SD 1.43) were achieved using a calculator and 70 % correct without a calculator (mean 7.19/ SD 2.34), the difference being statistically significant (*p* < 0.001). There was no statistically significant difference between the two groups on the use of calculator and results of the medication calculation test.

### Decision-making competence

In the patient vignettes, only four students chose all the preferable answers. On average, 57 % of the answers were correct when results from both groups were combined, higher values being observed among the 7th semester students than among the 2nd semester students (Additional file 5). In six of the patient vignettes there was only one correct answer, while in four there was also an additional second-best choice. The minimum acceptable score in the vignettes was therefore 10, with 14 points as the maximum. The minimum acceptable score was achieved by 84 % of the students (78 % of the 2nd semester students and 91 % of the 7th semester students). The difference between the groups was statistically significant (*p* < 0.001). With the exception of two vignettes, the students at the end of their education were significantly more able to solve the problems compared to 2nd semester students.

### Overall medication competence

To analyze overall medication competence, the results of the subcategories on medication competence were summarized. The average result in overall medication competence evaluation in was 70 % correct over the semesters; 68 % for the 2nd semester and 72 % for the 7th semester students (Table 2). The difference between the groups was statistically significant (*p* < 0.001).

**Table 2:** Results of the medication competence evaluation (% correct answers)

Test scores	Correct answers %(SD)		
	2nd semester (*n* = 327)	7th semester (*n* = 338)	Difference between the groups (*p*-value)
Theoretical medication competence Knowledge test (30 items)	71(14)	73(10)	ns
Practical medication competence Medication calculation test (10 items)	73 (24)	76 (22)	ns
Decision making competence Patients vignettes (10 items)	51 (22)	62 (18)	<0.001
Overall medication competence (50 items)	68 (13)	72 (10)	<0.001

*ns* no statistical difference, *SD* standard deviation, Statistical test: Mann–Whitney *U*-test

### Factors associated with nursing students’ medication competence

Most of the univariate factors explored this study were statistically associated with students’ medication competence. Among the individual factors older age (*p* = 0.007), previous good grade in mathematics (*p* = 0.01), good abilities of self-regulation in learning (*p* = 0.008), less lack of regulation in learning (*p* = 0.01), perception of pharmacology as easy (*p* = 0.002), participation in supportive education in medication calculations (*p* = 0.02), good self-confidence in medication management (*p* = 0.005) and good study motivation (*p* = 0.02), and among environmental factors, the use of the Medication Passport (*p* = 0.004) had a univariate correlation with the theoretical medication competence.

Among the individual factors, the univariate determinants of practical medication competence were older age (*p* = 0.0007), previous good grade in mathematics (*p* = 0.0007), long syllabus in mathematics in upper secondary school (*p* < 0.0001), participation in supportive education in medication calculations (*p* = 0.02), satisfaction with current medication education (*p* = 0.002), perception of mathematics and medication calculations as easy (*p* = 0.01), and among environmental factors, the use of a calculator (*p* < 0.0001). The univariate determinants of decision-making competence were, among individual factors, prior degree in nursing (*p* = 0.02), perception of pharmacology as easy (*p* = 0.009), less lack of regulation in learning (*p* = 0.009), and among the environmental factors, the 7th semester (*p* < 0.0001).

In multivariate regression analysis, nine individual factors and one environment factor were found to be independent determinants of the total medication competence evaluation (Table 3). We also cross-analyzed the sum-scores of medication competence areas and found them to be significantly interrelated (*p* < 0.001).

**Table 3:** The independent determinants of total medication competence (% of correct answers) (*n* = 594)

Determinant		n	Adjusted mean (SE) ^1)^	β (SE)	p ^2)^	IF/LEF
Age, years ^3)^					0.0002	IF
	19-20	86	67.7 (1.29)	−7.0 (1.74)		
	21-25	353	70.6 (0.69)	−4.1 (1.41)		
	26-30	85	73.1 (1.26)	−1.5 (1.71)		
	31-	70	74.6 (1.36)	0		
Long syllabus in matricular examination on mathematics					0.01	IF
	Yes	120	72.9 (1.12)	2.8 (1.15)		
	No	474	70.1 (0.63)	0		
Semester					0.03	LEF
	2nd semester	285	70.5 (0.77)	−2.1 (0.98)		
	7th semester	309	72.5 (0.93)	0		
Perception on pharmacology to be easy ^4)^					0.003	IF
	Agree	109	72.3 (1.17)	−0.4 (1.29)		
	Not agree, not disagree	241	69.5 (0.86)	−3.2 (0.98)		
	Disagree	244	72.7 (0.86)	0		
Satisfied on the amount of current education					0.04	IF
	Agree	179	71.3 (0.94)	−1.7 (1.01)		
	Not agree, not disagree	107	70.2 (1.16)	−2.8 (1.17)		
	Disagree	308	73.0 (0.78)	0		
Grade on mathematics		594	-	1.2 (0.59)	0.04	IF
Grade on exam on theoretical basis of medication management		594	-	1.2 (0.48)	0.01	IF
Self-Regulation in learning		594	-	1.4 (0.56)	0.01	IF
Lack of regulation in learning		594	-	−1.8 (0.62)	0.004	IF
Perception on mathematics and medication calculations to be easy		594	-	1.5 (0.54)	0.006	IF

*IF* individual factor, *LEF* learning environment factor

Model 100 xR-square = 19.4 %

Model F(14, 579) = 9.95, *p* < 0.0001

SE: standard error of estimate

^1)^ The adjusted mean is the mean value of the category adjusted by all other determinants in the model. ^2)^ Significance of the determinant. ^3)^ In pair-wise comparisons the following significant differences between categories were found: “19-20 y” and”26-30 y” (*p* = 0.008), “19-20 y” and “31- y” (*p* = 0.0004), “21-25 y” and “31- y” (*p* = 0.02). ^4)^ In pair-wise comparisons the following significant difference between categories was found: “Disagree” and”Not agree, not disagree” (*p* = 0.004)

Finally, we compared students in the lowest and highest quartile of medication competence (Table 4). In the highest quartile we found a positive association between students’ medication competence and long syllabus in mathematics, previous good grade in mathematics and exam in the theoretical basis of medication management, participation in supportive medication calculation education, perception of pharmacology and mathematics as easy, good study motivation, active participation in studying topics of medication care, good self-confidence in medication management, high abilities in self-regulated learning, less lack of regulation in learning, 7th semester and the number of clinical practice placements. Students’ previous academic success, such as a good grade in mathematics and exam in the theoretical basis of medication management, was a more significant factor associated with overall medication competence among the 2nd semester students (Table 4) whereas among the 7th semester students, students’ ability in self-regulated learning and study motivation were associated with their overall medication competence.

**Table 4:** Cross-evaluation on significance of associated factors in the two student groups based on the weakest and highest results on medication competence evaluation

Associated factor	Quartile difference in total	Quartile difference 2nd semester	Quartile difference 7th semester
Age	xx	ns	x
Gender	ns	x	ns
Previous degree in nursing	ns	ns	ns
Matricular exam in mathematics	xx	xx	
Long syllabus in mathematics	xxx	xx	
Failed medication calculation test	x	xx	x
Participation in supportive medication calculation education	xxx	xx	ns
Failed theoretical exam on basics of medication management	x	ns	ns
Perception of pharmacology easy	xxx	xx	x
Satisfaction on amount of current education	ns	ns	ns
High abilities of self-regulated learning	xxx	ns	xx
Less lack of regulation in learning	xxx	x	xx
Perceives mathematics as easy	xxx	xxx	xx
Good grade in mathematics	xxx	xxx	ns
Good grade on exam on theoretical basis of medication management	xxx	xxx	x
Good study motivation in studying medication care	xxx	ns	xxx
Active participation in studying medication care	xxx	x	xx
Self-confidence in medication management	xxx	xx	x
Semester	xxx	-	-
Possibility to apply theory in clinical practice (only 7th semester)	ns	-	ns
The Medication Passport in use	ns	ns	ns
The use of a calculator	x	ns	x
The number of clinical practice placements	xxx	ns	ns
Perceives the Medication Passport as useful (only 7th semester)	ns	-	ns
Work experience prior and during nursing education	ns	ns	ns

Statistical difference x =0.05, xx = 0.01, xxx = 0.001, ns = no statistical difference

## Discussion

### Medication competence of nursing students

Nursing students’ medication competence has been a concern for many years. However, as shown here, medication competence evolves during the education. The students were generally well-motivated and participated actively in medication education. In contrast to prior studies, the students in our study perceived themselves as self-confident in medication management at the end of their education [6, 7, 10, 16, 32].

Theoretical medication competence includes students’ knowledge base on subjects necessary for safe medication management, such as laws and regulations, pharmacology, pharmacy and medication administration. In our study, the theoretical medication competence of the students was better than in previous studies [6, 10, 11], but deficiencies still existed at the end of education. This is worrying, since theoretical medication competence is essential for safe medication care and necessary for making rational decisions. The difference between the two groups was statistically different only on sum score of medication package information and common abbreviations used in medication care, indicating limited improvement of theoretical medication competence during education. For nurse educators, our study results highlight the need to integrate the theoretical studies throughout the education and conduct regular evaluations during undergraduate education.

The practical medication competence of the students, as evaluated by medication calculation test, was rather good compared to several previous studies [7, 9–11, 16, 17, 19]. However, comparisons must be made with caution due to the different methods used in the evaluation. In Finland, the pass rate requirement on medication calculations is 100 % correct answers, and only 17 % of the students would have passed the test. This result verifies the need for continuing the development of medication calculation education and the need for regular evaluations [7, 10]. Practical medication competence also involves areas other than medication calculations, such as administering medicines correctly via different routes [38]. Further research is therefore warranted to explore students’ practical medication competence in authentic care or simulated situations of medication management. In order to improve the development of practical medication competence practically orientated, contextualized teaching and assessment methods and adequate possibilities to practice are necessary [4, 7, 10].

Decision-making integrates nursing students’ theoretical and practical medication competence. A sound knowledge base of pharmacology and medication management is required to understand and solve problems associated with patients’ treatment regimens in different care situations [4, 38, 39]. We introduced patient vignettes as an approach for medication competence evaluation. Patient vignettes provided us the possibility to evaluate students’ ability to integrate their knowledge base into decision-making. The results indicate a need for developing a deeper understanding of clinical pharmacology and patient education. However, students at the end of their education were significantly more able to make decisions, verifying the impact of education and clinical practice on medication competence. For nurse educators, the use of patient vignettes provides a possibility to contextualize medication care and management for the students. For future research, simulated patient vignettes in evaluation of medication competence with more qualitative methods could be useful.

### Factors associated with nursing students’ medication competence

Most of the factors associated with students’ medication competence were individual factors, and many of them, such as age, gender and semester, are beyond the influence of nurse educators. Students over 25 years old were able to achieve better results, as has also been found by Hutton [15] and McMullan et al. [9] in relation to numeracy skills. Our findings also verify the relationship between students’ mathematical success and the results in a medication calculation test [7, 15, 16, 18, 20], and support the arguments for a relationship between academic success and medication competence [13, 22], especially at the beginning of studies. Therefore, information on students’ previous academic success might be used to identify students in need of supportive education.

Students’ self-confidence had no explanatory value on students’ results in the medication calculation test as reported by Glaister [23], Andrew et al. [24], and McMullan et al. [25]. However, self-confidence in medication management was associated with better results in the knowledge test. Students who have better theoretical medication competence are also more self-confident in medication management. In future education, development of theoretical medication competence and confidence should therefore be supported. Thus, lack of confidence and feelings of anxiety may be more associated with fear of being tested [40, 41].

Nursing students are expected to be able to self-direct their learning, but they do not always use the time reserved for that in an efficient way. In our study, the students who were stronger in self-regulated learning achieved better results. This result supports the arguments on the importance of learning style in competence development [3, 30]. We also found a lack of regulation in learning to be associated with all areas of medication competence. Lack of regulation in learning indicates that students have difficulties to control the information load and self-direct their learning (35). Students with difficulties in learning due to information overload should be recognized [28]. Globally, the number of students with learning disabilities is increasing [42], and we need methods to identify them and to enhance their learning and competence development. In the future, it would be interesting to explore the relationships between nursing students’ learning disabilities, lack of regulation in learning, and medication competence.

The factors associated with students’ medication competence at the beginning of education are mainly related to prior and current academic success, but the impact is less significant at the end of the education. This finding verifies the results of Hutton [15] on the predictive value of previous mathematical grades prior to nursing education. Our results indicate that professional experience, high study motivation and ability to self-regulate learning explain prior graduation medication competence better than academic success. Practice possibilities in clinical learning environment are important for medication competence development, as has been highlighted also previously [6, 7, 32]. Application of a more health care environment sensitive instrument, such as the Health Care Learning and Studying Inventory (26), might be a good choice in further studies on factors associated with nursing students’ medication competence.

### Limitations

The present study has some limitations. The low response rate has to be observed when generalizing the results. The sample size, however, was based on power analysis and the calculated sample size was achieved. The sample also represented well students with different socio-demographic factors, polytechnic schools and the overall number of nursing students in Finland. The deficiencies in nursing students’ medication competence seem to be similar globally, and our results may therefore indicate the possibility of the same associated factors existing also among students from other countries. However, caution needs to be observed when generalizing the results outside Finland, since the education varies internationally, as does the registered nurse’s role and responsibility in medication management.

Also methodological limitations exists. Not all individual or learning environment factors can be explored in one study and by using a highly structured method. However, we have explored students’ medication competence from a broader perspective than in prior studies and have included most of the factors we identified in the prior literature review [21].

## Conclusion

Our study provided broad insight into nursing students’ medication competence and factors associated with it. The core elements of medication competence are significantly interrelated, highlighting the need to provide integrated and comprehensive medication education to support students’ competence development. To increase the safety of pharmacotherapy, there is a need for nursing education research that focuses on the effectiveness of teaching and learning methods that support the development of adequate medication competence. The deficiencies in medication competence identified in our study are similar globally, and therefore some solutions for developing medication education might be found by international research. Our study produced information for future nursing education research in this field, especially for developing study designs for evaluation of the effectiveness of teaching methods of students’ medication competence. However, safe medication practice is also dependent on many factors, such as organizational structures and policies, complexity of care processes, workload, and the environment where medications are dispensed. Therefore, further research using diverse research methodologies is also needed for a deeper understanding of nursing students’ medication competence.

The most significant association with students’ medication competence exists between students’ individual factors and medication competence. Students’ previous academic success is a more significant associated factor at the beginning of nursing education than at the end of it. In the 7th semester, students’ ability in self-regulated learning and study motivation are more significant factors. Methods to increase students’ abilities in self-regulated learning to manage the content-laden curricula and constantly expanding information load are therefore warranted. As the number of students with learning disabilities is increasing in undergraduate nursing cohorts, this presents yet another challenge to nurse educators globally. Therefore, further international research is required to investigate the link between reduced entry-level regulation in nursing and medication competence.

## Additional files

Additional file 1:
**Examples of the items used in medication competence evaluation.** (DOC 27 kb) Additional file 2:
**Educational background of the students.** (DOC 35 kb) Additional file 3:
**Results of the knowledge test (% correct answers).** (DOC 29 kb) Additional file 4:
**Results of the medication calculation test (% correct answers).** (DOC 39 kb) Additional file 5:
**Results of the patient vignettes (% of best actions chosen).** (DOC 34 kb)

## Abbreviations

ECTS: European credit transfer and accumulation system
MCAF: medication competence and associated factors - instrument
ILS: inventory of learning style - instrument [34]
