# Supplementary material for: Factors associated with nursing students’ medication competence at the beginning and end of their education
Source: BMC Med Educ. 2015 Dec 18;15:223. doi: 10.1186/s12909-015-0513-0 (PMC4683869; doi:10.1186/s12909-015-0513-0)
Supplement: Additional file 1: — Examples of the items used in medication competence evaluation. (DOC 27 kb) [file 12909_2015_513_MOESM1_ESM.doc]

*Additional file 1.* Examples of the items used in medication competence evaluation

| Theoretical medication competence | A drug is absorbed faster when administered in liquid rather than tablet form  (Right, Wrong, or I don’t know) |
| --- | --- |
| Practical medication competence | A physician has prescribed a child (weight 18 kg) Ermycin® (erythromycin) 40mg/kg/day divided into three doses. The strength of the oral solution is 80mg/mL. How many milliliters will you give the child in one dose? (1.5 mL, 2 mL, 2.5 mL, 3 mL, or I don’t know) |
| Decision-making competence  Patient vignette: You have an 82-year-old patient in home care who is on regular long-acting nitrate preparation, one tablet three times a day. The patient tells you during the home visit that she has forgotten to take her tablet in the morning. How do you act in this situation? | a) You advise the patient to take two tablets at the next medication time  b) You advise the patient not to take an extra tablet today but to continue with the normal dose  c) You advise the patient to take half a tablet more at the next medication time  d) I don’t know |
