# Supplementary material for: Factors associated with nursing students’ medication competence at the beginning and end of their education
Source: BMC Med Educ. 2015 Dec 18;15:223. doi: 10.1186/s12909-015-0513-0 (PMC4683869; doi:10.1186/s12909-015-0513-0)
Supplement: Additional file 2: — Educational background of the students. (DOC 35 kb) [file 12909_2015_513_MOESM2_ESM.doc]

*Additional file 2. E*ducational background of the students

| **Background factor** | **Nurse students both groups % (n)** | **2nd semester % (n)** | **7th semester % (n)** |
| --- | --- | --- | --- |
| ***Basic Education*** |  |  |  |
| Completed upper secondary school | 65 (481) | 80 (233) | 78 (243) |
| Long syllabus in mathematics in upper secondary school | 20 (130) | 22 (71) | 18 (59) |
| Short syllabus in mathematics in upper secondary school | 41 (276) | 41 (132) | 44 (144) |
| Not completed matriculation examination on mathematics | 19 (126) | 20 (64) | 19 (62) |
| Completed upper secondary, and diploma degree in nursing | 8 (51) | 9 (28) | 7 (23) |
| Diploma degree in nursing, not completed upper secondary school | 14 (91) | 13 (42) | 15 (49) |
|  |  |  |  |
| ***Success in previous studies*** |  |  |  |
| Last grade in mathematics prior to current education  Excellent  Good  Satisfactory  Mediocre | 22 (143)  41 (274)  32 (210)  5 (36) | 24 (78)  35 (115)  34 (110)  7 (23) | 19 (65)  47 (159)  30 (100)  4 (13) |
| Failed medication calculation at least one time | 64 (425) | 52 (171) | 76 (254) |
| Participated in supportive education on medication calculations | 20 (131) | 19 (63) | 20 (68) |
| Grade in examination on theoretical basis of pharmacotherapy (incl. principles of pharmacology, medication administration)  Excellent  Good  Satisfactory  Mediocre | 17 (113)  40 (266)  27 (181)  7 (47) | 21 (64)  38 (116)  25 (77)  10 (29) | 15 (49)  47 (150)  32 (104)  6 (18) |
