# Supplementary material for: Factors associated with nursing students’ medication competence at the beginning and end of their education
Source: BMC Med Educ. 2015 Dec 18;15:223. doi: 10.1186/s12909-015-0513-0 (PMC4683869; doi:10.1186/s12909-015-0513-0)
Supplement: Additional file 3: — Results of the knowledge test (% correct answers). (DOC 29 kb) [file 12909_2015_513_MOESM3_ESM.doc]

*Additional file 3.* Results of the knowledge test (% correct answers)

| ***Test scores*** | ***Correct answers %(SD)*** | |  |
| --- | --- | --- | --- |
|  | ***2nd semester***  ***(n=327)*** | ***7th semester***  ***(n=338)*** | ***Difference between the groups***  ***(p-value)*** |
| Legislation and guidelines in medication management (5 items) | 61(20) | 61(17) | ns |
| Medication package information and common abbreviations used in medication care (5 items) | 72(19) | 79 (15) | < 0.001 |
| Pharmacology (10 items) | 68(20) | 67(16) | ns |
| Handling and preparation of medicines and medication administration (10 items) | 78(17) | 81 (13) | ns |
| **Total knowledge test** (30 items) | 71(14) | 73(10) | ns |

ns = no statistical difference, SD=standard deviation, Statistical test: Mann-Whitney U-test
