# Supplementary material for: Factors associated with nursing students’ medication competence at the beginning and end of their education
Source: BMC Med Educ. 2015 Dec 18;15:223. doi: 10.1186/s12909-015-0513-0 (PMC4683869; doi:10.1186/s12909-015-0513-0)
Supplement: Additional file 4: — Results of the medication calculation test (% correct answers). (DOC 39 kb) [file 12909_2015_513_MOESM4_ESM.doc]

*Additional file* 4. Results of the medication calculation test (% correct answers)

| ***Medication calculation type*** | ***Correct answer*** *(%)* | | |
| --- | --- | --- | --- |
|  | ***2nd semester***  ***(n=327)*** | ***7th semester***  ***(n=336)*** | ***Difference between the groups***  ***p*** |
| Dosage (tablet, the sufficiency of medicine package) | 97 | 98 | ns |
| Dilution (dilution of oral liquid, percentage) | 29 | 37 | 0.026 |
| Pediatric dosage (oral liquid dosage) | 71 | 77 | ns |
| Dosage (conversion of oral liquid into tablet form) | 90 | 93 | ns |
| Dosage (injection) | 88 | 91 | ns |
| Dosage (per oral drops, the amount of medicine agent in one dose) | 78 | 85 | ns |
| Dosage (conversion of unit, tablet) | 79 | 81 | ns |
| Strength of liquid (conversion of percentage expression to mg/mL) | 63 | 78 | < 0.001 |
| Infusion rate (mL/h) | 84 | 85 | ns |
| Infusion rate (gtt/min) | 58 | 54 | ns |
| **Total medication calculation test** (10 items) Mean (SD) | 73 (24) | 76 (22) | ns |

ns = no statistical difference, SD=standard deviation, Statistical tests: Chi-Square, Mann-Whitney U-test
