# Supplementary material for: Factors associated with nursing students’ medication competence at the beginning and end of their education
Source: BMC Med Educ. 2015 Dec 18;15:223. doi: 10.1186/s12909-015-0513-0 (PMC4683869; doi:10.1186/s12909-015-0513-0)
Supplement: Additional file 5: — Results of the patient vignettes (% of best actions chosen). (DOC 34 kb) [file 12909_2015_513_MOESM5_ESM.doc]

*Additional file* 5. Results of the patient vignettes (% of best actions chosen)

| **Patient Vignette** | **Best action chosen (%)** | |  |
| --- | --- | --- | --- |
|  | **2nd semester students (n=323)** | **7th semester students (n=327)** | **Difference between the groups (p-value)** |
| Patient has forgotten to take her long-acting nitrate preparation medicine in the morning | 74 | 81 | 0.044 |
| Patient has warfarin medication and is in need of advice on pain relief | 60 | 81 | <0.001 |
| Patient has metronidatzole antimicrobial drug in use (advise on potential drug interaction with alcohol) | 52 | 60 | 0.041 |
| Patient has digoxin overdose symptoms | 41 | 56 | <0.001 |
| Patient has been prescribed a new medicine and he has lost the medicine package information | 50 | 57 | ns |
| Patient has hepatic insufficiency and is in need of advice on pain relief | 7 | 19 | <0.001 |
| One-month-old infant has a fever of 38.5°C, mother is in need of advice on the use of antipyretic medication for the infant | 52 | 51 | ns |
| Patient has symptoms of cerebral infarction, but the physician has ordered glucose intravenous liquid | 57 | 76 | p<0.001 |
| Patient has been prescribed a new medicine (beta blocker), but he forgot to tell his physician that he has asthma | 77 | 87 | 0.001 |
| Patient has insulin treatment for diabetes and he is going to gastroduodenoscopy. He is in need of advice about what to do with insulin in the morning of the procedure day. | 42 | 52 | 0.008 |
|  |  |  |  |
| Total score patients vignettes (10 items) | 51 (22) | 62 (18) | <0.001 |

ns = no statistical difference, SD=standard deviation, Statistical tests: Chi-square, Mann-Whitney U-test
